# Primary cilia and aberrant cell signaling in epithelial ovarian cancer

**DOI:** 10.1186/2046-2530-1-15

**Published:** 2012-08-10

**Authors:** Dorte L Egeberg, Mette Lethan, Robert Manguso, Linda Schneider, Aashir Awan, Tue S Jørgensen, Anne G Byskov, Lotte B Pedersen, Søren T Christensen

**Affiliations:** 1 Department of Biology, University of Copenhagen, Universitetsparken 13, 2100, Copenhagen, Denmark; 2 Laboratory of Reproductive Biology, University Hospital of Copenhagen, Blegdamsvej 9, 2100, Copenhagen, Denmark

## Abstract

**Background:**

Ovarian cancer is the fourth leading cause of cancer-related deaths among women in Denmark, largely due to the advanced stage at diagnosis in most patients. Approximately 90% of ovarian cancers originate from the single-layered ovarian surface epithelium (OSE). Defects in the primary cilium, a solitary sensory organelle in most cells types including OSE, were recently implicated in tumorigenesis, mainly due to deregulation of ciliary signaling pathways such as Hedgehog (Hh) signaling. However, a possible link between primary cilia and epithelial ovarian cancer has not previously been investigated.

**Methods:**

The presence of primary cilia was analyzed in sections of fixed human ovarian tissue as well as in cultures of normal human ovarian surface epithelium (OSE) cells and two human OSE-derived cancer cell lines. We also used immunofluorescence microscopy, western blotting, RT-PCR and siRNA to investigate ciliary signaling pathways in these cells.

**Results:**

We show that ovarian cancer cells display significantly reduced numbers of primary cilia. The reduction in ciliation frequency in these cells was not due to a failure to enter growth arrest, and correlated with persistent centrosomal localization of aurora A kinase (AURA). Further, we demonstrate that ovarian cancer cells have deregulated Hh signaling and platelet-derived growth factor receptor alpha (PDGFRα) expression and that promotion of ciliary formation/stability by AURA siRNA depletion decreases Hh signaling in ovarian cancer cells. Lastly, we show that the tumor suppressor protein and negative regulator of AURA, checkpoint with forkhead-associated and ring finger domains (CHFR), localizes to the centrosome/primary cilium axis.

**Conclusions:**

Our results suggest that primary cilia play a role in maintaining OSE homeostasis and that the low frequency of primary cilia in cancer OSE cells may result in part from over-expression of AURA, leading to aberrant Hh signaling and ovarian tumorigenesis.

## Background

Epithelial ovarian cancer (EOC) belongs to a heterogeneous group of neoplasms that exhibit a wide range of molecular defects, affecting cell survival, proliferation, differentiation and migration. EOC is the most lethal of the gynecologic malignancies, accounting for more than 90% of all ovarian malignancies, and is mainly a disease of postmenopausal women [1]. The high mortality rate of EOC is primarily due to difficulties in diagnosing early stages of the disease. Most patients (approximately 75%) present with advanced stage (III/IV) tumors, for which the five-year survival rate is below 46% [1]. This is not surprising given the size and location of the ovaries, making them not readily accessible by pelvic examination unless significantly enlarged. Improvements in surgical techniques and chemotherapy regiments over the last three decades have resulted in improvements in ovarian cancer treatment; however, despite these advances most patients treated for EOC eventually develop disease recurrence [2,3].

The etiology behind EOC is poorly understood, although invagination clefts and inclusion cysts lined with ovarian surface epithelium (OSE) have been pointed out as hot spots for initiation of neoplastic processes in EOC [4-6]. Further, a number of recent studies have indicated that EOC is linked to aberrant cell signaling, including Hedgehog (Hh) and platelet-derived growth factor (PDGF) signaling as well as over-expression of aurora A kinase (AURA) and deregulated expression of the novel tumor suppressor protein, checkpoint with forkhead-associated and ring finger domains (CHFR) [7-19]. Consequently, targeted agents against Hh pathway components, PDGFR and AURA have been explored recently in the management of ovarian cancer and recurrent disease [20].

Hh signaling regulates cell proliferation and differentiation in numerous tissues during embryonic and fetal development and remains active in the adult body where it is involved in the maintenance of stem cell populations [21-23]. Hh signaling depends on a fine-tuned intracellular signal mediated by the repressor or activator forms of the transcription factors GLI2 and GLI3, and is mainly based on a positive feedback loop via *GLI1* and a negative feedback loop via *Patched-1* (*PTCH1*) transcription [24,25]. It is, in particular, these feedback loops that are found disturbed in EOC specimens [7-10].

PDGFR signaling regulates cell growth and survival, transformation, migration and wound healing [26]. Several reports document a change in the expression level of the alpha form of PDGFR (PDGFRα) compared to normal OSE cells and that this expression is associated with high tumor grade, high proliferation index, and poor survival rate [11-14].

AURA is a major mitotic kinase involved in centrosome maturation, mitotic entry, and spindle assembly [27]. AURA maps to a chromosomal region frequently shown to be amplified in human ovarian cancer [15,16,18], and several studies have identified elevated AURA kinase activity and/or increased protein level as common characteristics in ovarian cancer [15-17,28].

CHFR is a novel player in the genesis and progression of EOC [19]. CHFR has multiple functions in checkpoints during mitosis, such as regulation of the G2/M transition by its inherent ubiquitin ligase activity and targeting of key proteins, such as AURA, to the proteasome [29-32]. Nevertheless, a better understanding of the multiple signaling pathways associated with ovarian tumorigenesis is needed in order to identify new ways to target signaling pathways in EOC and in this way increase the efficiency of ovarian cancer treatment and minimize recurrent disease.

Recent research showed that primary cilia may play a critical role in tumorigenesis and cancer progression by functioning as a tumor suppressor organelle that regulates cell proliferation, differentiation, polarity, and migration [33,34]. Primary cilia are microtubule-based organelles emanating from the distal end of the mother centriole located beneath the plasma membrane during growth-arrest [35]. Reception and transduction by the cilium of chemical and mechanical signals from the extracellular environment is made possible by specific receptors and ion channels located in or near the ciliary membrane. Here signaling pathways regulated by receptor tyrosine kinases, G-protein-coupled receptors, notch receptors, receptors for extracellular matrix proteins and TRP ion channels, including Hh, Wnt and PDGFRα signaling [35-39], are coordinated. The functional importance of the primary cilium is reflected by a number of severe genetic diseases and developmental disorders caused by dysfunction of cilia, commonly referred to as ciliopathies [40,41]. Recent studies have associated some cancers with loss of primary cilia resulting in deregulated cell proliferation, and others with deregulated ciliary signaling [42-49]. As an example, Wong *et al*. [46] demonstrated a role of the primary cilium as an important modulator of Hh signaling in basal cell carcinoma development. They showed that loss of primary cilia in mouse skin cells with a constitutive active Gli2 accelerated tumorigenesis due to disruption in Gli2/Gli3 processing, leading to an altered Gli2 activator/Gli3 repressor ratio . Furthermore, over-expression of an activated form of GLI2 was shown to activate Hh target genes in two prostate cancer cell lines without primary cilia, while over-expression of an activated form of Smoothened (SMO) was not [47,50]. Cilium resorption can occur as a physiological consequence of cell cycle progression, but, as outlined above, any alteration in physiological ciliary formation or function can have disastrous effects. Interestingly, AURA, which is found to be highly over-expressed in a variety of human cancers [18,51-53], was recently proposed to regulate disassembly of primary cilia upon mitogenic stimulation [54]. The proposed molecular mechanism includes co-localization of AURA and the scaffolding protein HEF1 at the ciliary basal body and subsequent phosphorylation and activation of the tubulin deacetylase HDAC6, leading to destabilization and resorption of the ciliary axoneme [54]. Although AURA is frequently over-expressed or deregulated in human ovarian cancer cells [15-18,28], it is unknown whether this correlates with defective primary cilia in these cells.

In this report, we investigated the occurrence of functional primary cilia in growth-arrested normal human OSE cells and two different human ovarian adenocarcinoma cell lines (SK-OV3 and OVCAR3; referred to in the text as cancer OSE cell lines) with the focus on the correlation between centrosomal AURA levels and the presence or absence of cilia and cilia-related signaling pathways. We show that the majority (>60%) of normal growth-arrested OSE cells display primary cilia with PDGFRα and Hh signaling components. In contrast, the fraction of growth-arrested cancer OSE cells with primary cilia was less than 20%, and these cells displayed aberrant Hh signaling and down-regulated expression and/or glycosylation of PDGFRα. We also show that AURA is up-regulated in cancer OSE cells and that RNAi-induced depletion of AURA in these cells leads to a modest, but significant, increase in the number of ciliated cells and partial restoration of Hh signaling. Finally, we show that CHFR localizes to the ciliary basal body in OSE cells. These results suggest that primary cilia play a role in maintaining OSE homeostasis and that the low frequency of primary cilia in cancer OSE cells may result in part from over-expression of AURA, leading to aberrant Hh signaling and ovarian tumorigenesis.

## Results

### Characterization and isolation of human OSE cells in cultures

**Figure 1:**
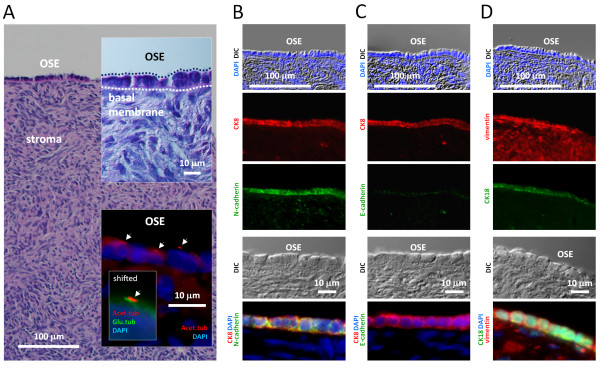
**Characterization of ovarian surface epithelium. A**) Section through a normal adult ovarian cortex stained with H & E, showing OSE cells at top as a single layer, separated from the underlying stroma by a basal membrane (dotted line). The inserts show OSE cells in higher magnification, and primary cilia (arrows) on OSE cells visualized by IFM analysis with antibodies against acetylated α-tubulin (Acet.tub) and detyrosinated α-tubulin (Glu.tub). **B-D**) IHC analysis of ovarian tissue. OSE cells are characterized by being positive for the epithelial markers cytokeratin 8 (CK8) and cytokeratin 18 (CK18), and for the mesenchymal markers N-cadherin and vimentin, whereas they are E-cadherin negative. Nuclei are visualized with DAPI. DAPI, 4',6-diamidino-2-phenylindole; IFM, immunofluorescence microscopy; IHC, immunohistochemistry; OSE, ovarian surface epithelium.

To study a potential link between ovarian cancer and defective primary cilia, we first used immunohistochemistry (IHC) to characterize primary cilia in OSE cells of tissue sections from healthy human ovaries (see Methods for details). Consistent with previous observations [55], we found that OSE is a one-layered epithelium of squamous to cuboidal cells resting on a basal membrane covering the ovaries (Figure 1A). OSE cells possess primary cilia, as visualized with antibodies against two well-characterized ciliary markers, acetylated α-tubulin (Acet.tub) and detyrosinated α-tubulin (Glu.tub) (Figure 1A, lower insert) as described earlier [56]. Further, consistent with previous observations [57-59], we found that OSE has a limited commitment to the epithelial phenotype indicated by the retention of mesenchymal features, such as expression of vimentin and N-cadherin, and it lacks some of the typical epithelial characteristics, such as E-cadherin (Figure 1B-D).

**Figure 2:**
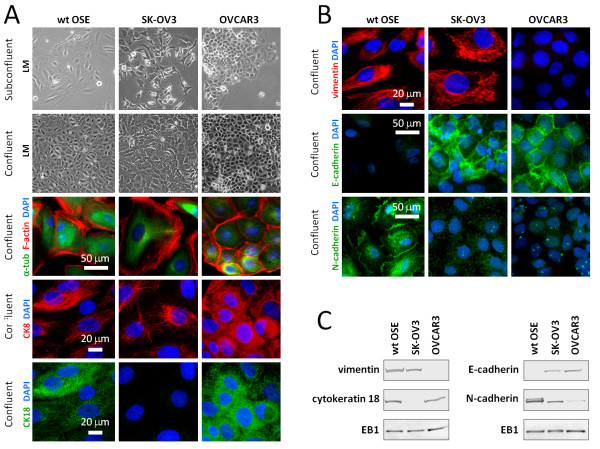
**Characterization of cell cultures of ovarian surface epithelium. A**) Light microscopy (LM) images of wt and cancer OSE cells in cultures at sub-confluent and confluent stages. Anti-α-tubulin (α-tub) and phalloidin (F-actin) were applied to vizualize the cytoskeletal microtubules and actin, respectively, by IFM analysis, and anti-cytokeratin 8 (CK8) and anti-cytokeratin 18 (CK18) were applied to visualize the keratin components characteristic of human OSE cells. **B**) IFM analysis with primary antibodies against markers differentially expressed in wt and cancer OSE cells. See text for details. DNA was stained with DAPI. **C**) WB of wt and cancer OSE cells showing their expression of proteins characteristic for human OSE cells. EB1 was applied as loading control. DAPI, 4′,6-diamidino-2-phenylindole; IFM, immunofluorescence microscopy; OSE, ovarian surface epithelium; WB, western blot; wt, wild type.

The active engagement of OSE cells in tissue repair is in keeping with the dual epithelial-mesenchymal phenotype or uncommitted phenotype, characterized by co-expression of cytokeratins and vimentin (Figure 1B-D), which likely confers advantages during the postovulatory repair of the ovarian surface *in vivo*. Furthermore, OSE differs from other epithelia by its tenuous attachment to its basement membrane, from which it is easily detached by mechanical means [60]. We exploited this feature of OSE to establish primary cell cultures of OSE cells by gentle scraping of the ovarian surface. We refer to these cells here as wild type (wt) OSE cells. In the light microscope wt OSE cells appear almost cubic and are organized in a regular pavement with close intercellular apposition and no overlap in the confluent stage (Figure 2A). We also analyzed cultures of SK-OV3 and OVCAR3, which are well-described human ovarian adenocarcinoma cell lines [61,62] often used as experimental models for EOC. In line with the heterogeneity of ovarian neoplasms, the morphologies of the two cancer OSE cell lines are very unlike each other; OVCAR3 forms a cobblestone-like monolayer with foci of multi-layering at high cell densities. In the subconfluent stage, the cells grow in small colonies separated by void spaces, which over time become sparse due to expansion of the colonies. OVCAR3 cells are epithelial in morphology, whereas SK-OV3 cells are atypical and more fibroblast-like with long and slender cells. In the confluent stage, SK-OV3 cells form a monolayer with a more disordered appearance compared to the wt OSE and OVCAR3 cells (Figure 2A). Even though the morphologies of the two cancer OSE cell lines differ they are both of epithelial origin [62,63].

We next characterized the wt and cancer OSE cell lines using antibodies specific for different cytoskeletal proteins and OSE markers in immunofluorescence microscopy (IFM) and western blot (WB) analysis. Consistent with IHC analysis of OSE in ovarian tissue sections (Figure 1B-D), cultured wt OSE cells were positive for cytokeratin-8 and −18 (CK8 and CK18) as well as vimentin and N-cadherin, and negative for E-cadherin (Figure 2A-C). In contrast, only a very few CK8 and CK18 positive cells were observed in the SK-OV3 cell line, whereas OVCAR3 cells were positive for both. However, OVCAR3 cells were negative to vimentin staining. Furthermore, both cancer cell lines expressed E-cadherin, which localized to the cell borders, whereas anti-N-cadherin stained a punctuated material within the cells (Figure 2A-C). These findings correlate well with previous reports indicating that ovarian cancer cells display a more classical epithelial phenotype compared to normal OSE cells [59,64,65].

### Reduced frequency of primary cilia in cultures of human cancer OSE cells

**Figure 3:**
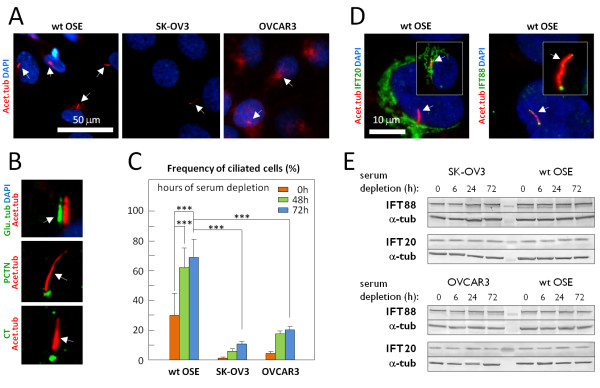
**Characterization of primary cilia formation in OSE cell cultures.** Wild type (wt) and cancer OSE cells were grown to confluence followed by serum depletion for 72 hours to induce growth arrest and formation of primary cilia (arrows). Microtubules of the ciliary axoneme were detected with anti-acetylated α-tubulin (Acet.tub) and anti-detyrosinated α-tubulin (Glu.tub) in IFM analysis (**A**, **B**). The pericentriolar material of the centrosome and the centrioles were visualized with anti-pericentrin (PCTN) and anti-centrin (CT) antibodies, respectively. **C**) Cilia frequencies were determined, by IFM analysis with anti-Acet.tub and/or anti-Glu.tub, antibodies, as the number of ciliated cells over the total cell number in sub-confluent cultures in the presence of serum (0 hour) and in confluent cultures serum-depleted for 48 or 72 hours. Error bars represent standard deviations. Data were tested for significance using one-way ANOVA. The level of significance was set at *P* < 0.001 (***). **D**) Localization of IFT20 and IFT88 was visualized by IFM in serum-depleted wt OSE cultures. Nuclei were stained with DAPI. **E**) WB analysis of IFT88 and IFT20 in wt and cancer OSE cells in sub-confluent cultures with serum (0 hour) and confluent cultures depleted for serum for six, 24, or 72 hours. α-Tubulin (α-tub) was applied as loading control. ANOVA, analysis of variance; DAPI, 4′,6-diamidino-2-phenylindole; IFM, immunofluorescence microscopy; OSE, ovarian surface epithelium; WB, western blot; wt, wild type.

Primary cilia emerge from OSE cells in tissue sections of mouse and human ovaries [56], but the function of these cilia remains to be investigated (Figure 1A). To address this we first investigated the occurrence of primary cilia in cultures of wt and cancer OSE cells using IFM with antibodies against ciliary (Acet.tub, Glu.tub) and centrosomal (pericentrin, centrin) markers. IFM was performed on sub-confluent cultures grown in the presence of serum (0 hour) and in confluent cultures that had been serum-starved for 48 or 72 hours to induce formation of primary cilia, and the number of ciliated cells for each growth condition was quantified (Figure 3A-C). The results show that both wt and cancer OSE cells possess primary cilia and that serum depletion increases the frequency of ciliated cells in the cultures (Figure 3C). However, both cancer OSE cultures have significantly fewer ciliated cells compared to wt OSE cultures, even after 72 hours of serum starvation (Figure 3C).

Construction of the ciliary axoneme requires intraflagellar transport (IFT), a bidirectional transport system driven by motor protein complexes that bring axonemal precursors to the growing tip of the cilium and return turnover products to the base [66]. Since IFT20 and IFT88 are required for effective ciliogenesis [67-69], we assessed the sub-cellular localization and expression of these proteins in the wt and OSE cell cultures grown in the presence or absence of serum. Similar to findings in other cell types [67-69], IFT20 was localized at the Golgi apparatus and IFT88 at the base and tip of primary cilia in wt OSE cells (Figure 3D). Further, WB analysis of lysates from wt and cancer OSE cell cultures grown with or without serum demonstrated that IFT88 and IFT20 are expressed at similar levels in all three OSE cell lines (Figure 3E). Thus the reduced frequency of ciliated cells in cancer OSE cells is unlikely to result from lack of these IFT proteins.

**Figure 4:**
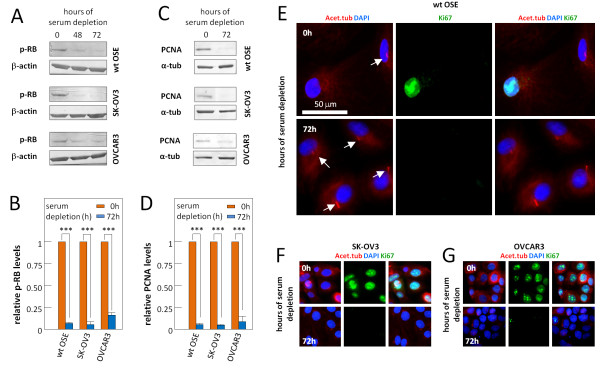
**wt and cancer OSE cells enter growth arrest upon serum depletion.** Analysis of known cell cycle markers in sub-confluent cultures with serum (0 hour) and confluent cultures serum depleted for 48 and/or 72 hours. **A, B**) WB analysis showing protein expression of phosphorylated retinoblastoma protein (p-RB) in wt and cancer OSE cells and quantification of p-RB levels in serum depleted cultures relative to cultures with serum and with respect to the loading controls (β-actin). **C, D**) WB analysis showing protein expression of proliferating cell nuclear antigen (PCNA) in wt and cancer OSE cells and quantification of PCNA levels in serum depleted cultures relative to cultures with serum and with respect to the loading controls (α-tub). **E-G**) IFM analysis of wt and cancer OSE cells using anti-acetylated α-tubulin (Acet.tub) antibody to detect primary cilia (arrows), and anti-Ki67 antibody to visualize Ki67 expression of cycling cells. Nuclei were visualized with DAPI. Note that most cancer cells enter growth arrest upon serum starvation, as judged by the lack of nuclear Ki67 staining. DAPI, 4′,6-diamidino-2-phenylindole; IFM, immunofluorescence microscopy; OSE, ovarian surface epithelium; WB, western blot; wt, wild type.

Primary cilia are normally absent in rapidly proliferating cells due to their resorption during cell cycle progression [35,66]. Therefore, we speculated whether the low frequency of primary cilia in cancer OSE cells results from a high rate of proliferation. To address this, we analyzed the wt and cancer OSE cells by WB and IFM using antibodies against known cell proliferation markers. As shown in Figure 4A-D, wt OSE cells are able to enter growth arrest after 72 hours of serum depletion, as indicated by an approximate 12-fold reduction in the level of phosphorylated retinoblastoma protein (p-RB) and an approximate 17-fold reduction in the level of proliferating cell nuclear antigen (PCNA). Similar results were obtained for SK-OV3 cells whereas the reductions in p-RB and PCNA levels in OVCAR3 cells were slightly less prominent with reductions of about six-fold and 11-fold, respectively (Figure 4A-D). IFM analysis with Ki67 antibody confirmed that both wt OSE and SK-OV3 cells are able to enter growth arrest upon serum depletion (Figure 4E,F), whereas the ability of the OVCAR3 cells to enter growth arrest seems to be slightly compromised (Figure 4G). Taken together, our results (Figures 3 and 4) indicate that the majority of growth-arrested wt OSE cells possess primary cilia, whereas the majority of growth-arrested cancer OSE cells do not. The results further suggest that the reduced frequency of primary cilia in cancer OSE cells is not primarily due to the inability of these cells to enter growth arrest.

### Hedgehog and PDGFRα signaling are associated with OSE primary cilia and are disrupted in cancer OSE cells

**Figure 5:**
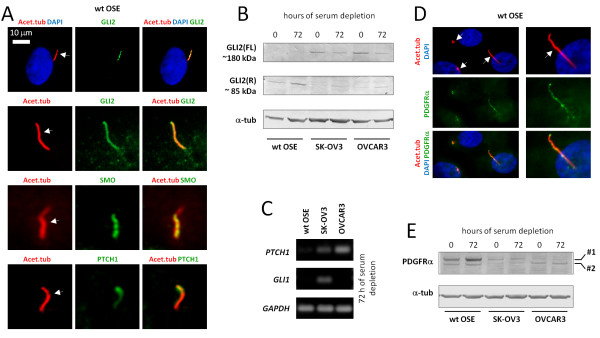
**Hedgehog components and PDGFRα localize to OSE primary cilia.** IFM analysis of serum-starved wt OSE cells using antibodies against the Hh components GLI2, SMO, and PTCH1 (**A**), or PDGFRα (**D**). Primary cilia (arrows) were detected with anti-acetylated α-tubulin (Acet.tub) antibody. **B**) WB analysis of wt and cancer OSE cells grown in the presence (0 hour) and or absence (72 hours) of serum. Blots were probed with antibodies against the full-length activator form of GLI2, GLI2(FL), or the C-terminally processed repressor form of GLI2, GLI(R). α-tubulin (α-tub) was applied as loading control. **C**) RT-PCR showing the expression level of the Hh responsive genes *PTCH1* and *GLI1* in cultures of wt and cancer OSE cells serum depleted for 72 hours. *GAPDH* was applied as an internal control. **E**) WB analysis of wt and cancer OSE cells grown in the presence (0 hour) or absence (72 hours) of serum. The PDGFRα antibody used recognizes two protein bands; #1 is the fully glycosylated form and #2 is the partly glycosylated form of the receptor. α-tub was applied as loading control. IFM, immunofluorescence microscopy; Hh, hedgehog; OSE, ovarian surface epithelium; WB, western blot; wt, wild type.

As described in the Background, Hh and PDGFR signaling have both been associated with ovarian tumorigenesis. Since Hh signaling components and PDGFRα localize to primary cilia in a variety of cell types [37], we investigated if primary cilia are associated with Hh and PDGFRα signaling in wt and cancer OSE cells. IFM analysis with antibodies against different Hh components showed that GLI2, SMO and PTCH1 localize to primary cilia in wt OSE cells (Figure 5A). Since processing of the full-length GLI2 (activator form) to the repressor form is known to depend on primary cilia [70-72], we explored GLI2 processing in wt and cancer OSE cells. WB analysis showed that wt OSE cells grown in the presence or absence of serum contain no or very little full-length GLI2 (activator) under either culture condition, whereas the processed repressor form of GLI2, GLI2(R), is present (Figure 5B). In contrast, sub-confluent, non-starved cultures (0 hour) of both cancer OSE cell lines contained a higher level of full-length and a much lower level of the GLI2 repressor form compared to wt OSE cells (Figure 5B). After 72 hours of serum starvation, the level of full-length GLI2 decreased in the OSE cancer cell lines and the level of repressor GLI2 increased, but not to levels comparable to those of wt OSE cells (Figure 5B). Consistent with these results, RT-PCR analysis in serum-starved cultures demonstrated that even in the absence of added Hh ligands to the medium, the SK-OV3 cells have a higher basal transcription of Hh responsive genes (*PTCH1, GLI1*) compared to wt OSE cells, whereas OVCAR3 only has a higher basal transcription of *PTCH1* (Figure 5C). The different expression patterns of Hh target genes in ovarian cancer cell lines might reflect the heterogeneity of ovarian neoplasms as postulated by others (see above). Thus, the cancer OSE cells display increased basal expression of Hh responsive genes.

We next investigated the localization and expression of PDGFRα in OSE cultures by IFM and WB analysis. PDGFRα was previously shown to be up-regulated during growth arrest [73] and to localize to primary cilia in fibroblasts [74,75] and other cell types [36]. However, ciliary localization of PDGFRα has not previously been reported for OSE cells. As shown in Figure 5D, we found that PDGFRα localizes to primary cilia of wt OSE cells and PDGFRα is up-regulated during growth arrest in these cells (Figure 5E). In contrast, SK-OV3 and OVCAR3 cells display a markedly lower level of PDGFRα protein and no increase in PDGFRα level is observed upon serum depletion in these cell lines (Figure 5E). As described elsewhere [74], the PDGFRα antibody used recognizes two protein bands in WB analysis; a high-molecular weight protein band representing the mature and fully glycosylated form and a low-molecular weight protein band representing the immature and only partly glycosylated form of the receptor. Notice that in OVCAR3 cells only the low-molecular weight form of the receptor (#2) is detectable in WB analysis (Figure 5E). These data indicate that PDGFRα signaling via primary cilia during growth arrest likely is perturbed in cancer OSE cells, although this requires further investigations.

### The level of aurora A kinase is reduced at the ciliary base in normal OSE cells and up-regulated in cancer OSE cells with defective primary cilia

**Figure 6:**
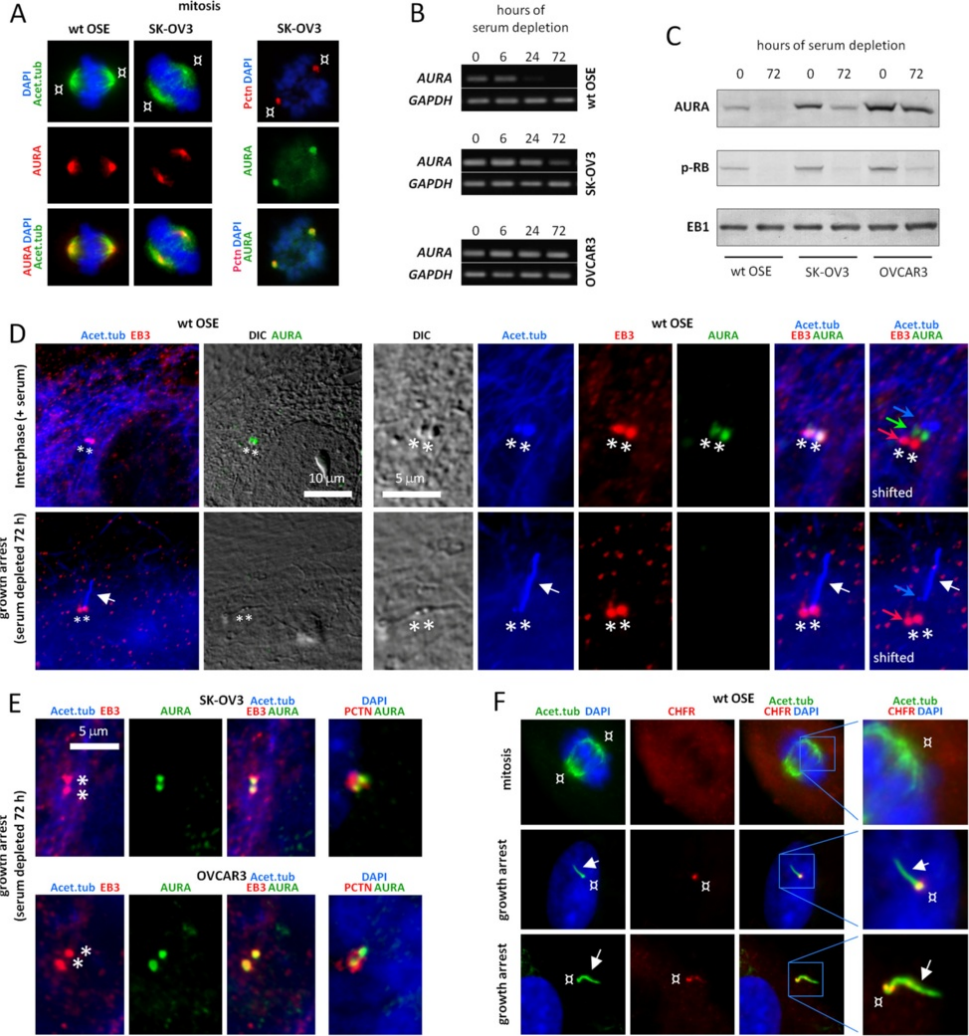
**Aurora A localization and expression in wt and cancer OSE cells.** IFM analysis of mitotic (**A**), interphase (**D**), and growth-arrested (**D, E**) wt and cancer OSE cells. Anti-pericentrin (PCNT), anti-EB3, anti-acetylated α-tubulin (Acet.tub), and anti-AURA were applied to detect the pericentriolar material (¤), centrioles (*), mitotic spindle and primary cilia (white arrows), and AURA, respectively. The rightmost column in (**D**) is a shifted overlay of the Acet.tub, AURA, and EB3 stainings. Cells in **A,** and rightmost column in **E** were fixed in mix-fix (see Methods for details), and cells in (**D, E**) were fixed in PFA + MeOH fix. **B**, **C**) AURA mRNA and protein levels were analyzed with RT-PCR (**B**) and WB (**C**), respectively, in sub-confluent cells with serum (0 hour) and in confluent cultures serum-depleted for 72 hours. *GAPDH* (**B**) and EB1 (**C**) were used as controls. Anti-phosphorylated retinoblastoma protein (p-RB) was included in WB analysis to verify that starved cells were in growth arrest (**C**). **F**) IFM analysis of checkpoint with forkhead-associated and ring finger domains (CHFR) localization in wt OSE cells during mitosis and in growth-arrested, serum-depleted cells. Cells were fixed in mix-fix and stained with antibodies as indicated. Primary cilia are marked with arrows and centrioles/basal bodies are marked with ¤. DNA was stained with DAPI. DAPI, 4′,6-diamidino-2-phenylindole; IFM, immunofluorescence microscopy; OSE, ovarian surface epithelium; WB, western blot; wt, wild type.

AURA has been implicated in cilia disassembly in *Chlamydomonas*[76] and mammalian cells [54]. This protein is often over-expressed in ovarian cancer cells. Therefore, we speculated whether the reduced frequency of ciliated cells observed in cancer OSE cells (Figure 3C) is linked to altered protein level and/or localization of AURA compared to wt OSE cells. To investigate this we analyzed the localization and expression of AURA in OSE cells by IFM, RT-PCR and WB. During mitosis, AURA displayed a centrosomal localization in both wt and cancer OSE cells (Figure 6A), consistent with previous reports [77]. Upon induction of growth arrest by serum depletion, the expression level of AURA was decreased dramatically in wt OSE cells, both at the mRNA (Figure 6B) and protein level (Figure 6C). AURA levels in serum-depleted SK-OV3 cells were reduced compared to non-starved cells but not to the same extent as serum-starved wt OSE cells. In contrast, AURA levels in serum-depleted OVCAR3 cells were largely similar to those of non-starved cells and significantly higher than those of starved wt OSE cells (Figure 6B, C). The elevated cellular level of AURA in the serum-depleted cancer OSE cells was not caused by a failure of these cells to enter growth arrest, as judged by WB with p-RB antibody (Figure 6C).

Consistent with the results of WB and RT-PCR analyses (Figure 6B,C), IFM analysis revealed that the centrosomal pool of AURA was clearly diminished in serum-depleted, ciliated wt OSE cells compared to non-starved cells (Figure 6D). Similarly, we observed that centrosomes of the few ciliated cancer OSE cells lacked AURA (data not shown). However, in serum-depleted SK-OV3 and OVCAR3 cells, centrosomes (marked with anti-EB3 and anti-pericentrin) mostly lacked primary cilia (stained with anti-Acet.tub) and were clearly AURA positive (Figure 6E; see also Figure 3C). The over-expression and localization of AURA to centrosomes in growth-arrested cancer OSE cells suggest that AURA may play a role in suppressing ciliogenesis and/or promoting ciliary disassembly in cancer OSE cells.

### The tumor suppressor protein, CHFR, localizes to the base of OSE primary cilia

In the mouse, the tumor suppressor protein, Chfr, is known to inhibit AurA by ubiquitination and proteasomal degradation [32]. The potential involvement of AURA in regulating cilia assembly or disassembly in human OSE cells (see above) prompted us to investigate whether CHFR is associated with the centrosome/cilium axis in these cells. To this end, we generated a polyclonal rabbit antibody against human CHFR (see Methods for details). WB analysis of lysates of cultured, serum-starved hTERT-RPE1 or NIH3T3 cells demonstrated that the CHFR antiserum recognizes a single band of about 73 kDa equivalent to the predicted size of endogenous CHFR (73.4 kDa for isoform 1) (Additional file 1: Figure S1A, B), and by WB analysis the CHFR antibody also recognized exogenous green fluorescent protein (GFP)-tagged CHFR expressed stably in serum-starved hTERT-RPE1 cells (Additional file 1: Figure S1C). Further, both endogenous and CHFR and GFP-tagged CHFR localized to the base of primary cilia in serum-starved hTERT-RPE1 cells (Additional file 1: Figure S1D, E). In serum-starved wt OSE cells the CHFR antibody predominantly labeled the base of primary cilia, but no clear localization of the antibody was observed in interphase or mitotic wt OSE cells (Figure 6F). However, in hTERT-RPE1 cells CHFR was detected at centrosomes in growth-arrested as well as cycling cells (data not shown), suggesting that the lack of detection of CHFR at centrosomes of mitotic OSE cells could be due to low abundance of the protein. These data conflict with previous studies showing that over-expressed, epitope-tagged CHFR displays a predominantly nuclear localization [78-80], but are in agreement with studies showing that endogenous CHFR localizes to cytoplasm and centrosomes during interphase growth [31,81,82] and to spindle poles during mitosis [82]. This is the first report on CHFR localization to primary cilia, and future studies might reveal if CHFR takes part in the signaling machinery that regulates ciliary disassembly.

### Depletion of AURA increases the frequency of primary cilia and reduces Hh signaling in cancer OSE cells

**Figure 7:**
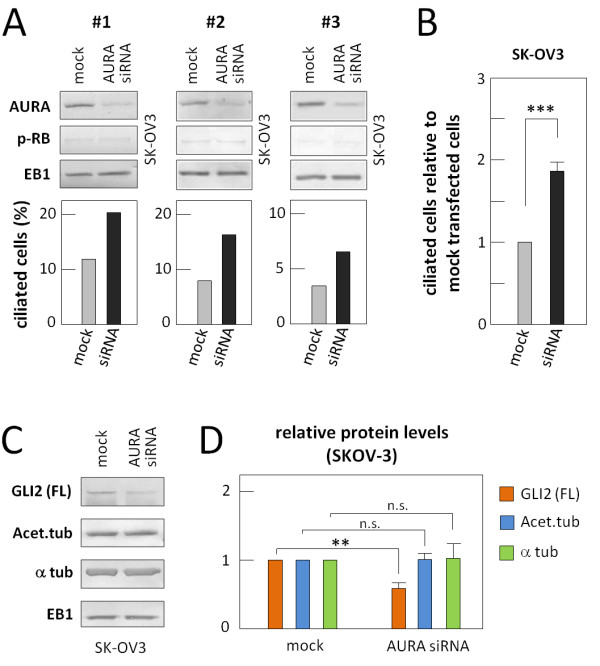
**Inhibition of Aurora A and effect on ciliary formation in cancer OSE cells. A**) Results of three independent siRNA knock-down experiments of AURA in SK-OV3 cells. Cells were grown to 60% confluency and transfected with scrambled oligonucleotides (mock) or siRNA targeting *AURA* mRNA. The cells were allowed to grow to confluence and 24 hours after transfection, medium was changed to serum-depleted medium. After 72 hours of incubation in serum-depleted medium, AURA protein levels were examined by WB analysis. Anti-phosphorylated retinoblastoma protein (p-RB) was included to explore the cell cycle stage. EB1 was applied as loading control. Percentages of ciliated cells were determined by IFM analysis with primary antibodies against acetylated α-tubulin, detyrosinated α-tubulin, and DAPI nuclear staining as the number of ciliated cells over the total cell number. Error bars represent standard deviations. **B**) Bar graph representation of the level of ciliated SK-OV3 cells in AURA siRNA transfected cells relative to mock transfected cells. The percentage of ciliated cells in mock transfected cells was set to one. Data were tested for significance using Student’s t-test. The level of significance was set at *P* < 0.001 (***). **C**) Protein levels of the full length activator form of GLI2, GLI2 (FL), acetylated α-tubulin (Acet.tub), and α-tubulin (α-tub) in mock and AURA siRNA transfected SK-OV3 cells serum-depleted for 72 hours. **D**) Bar graph representation of the relative protein levels in AURA siRNA transfected SK-OV3 cells compared to mock transfected cells. The protein level in mock transfected cells was set to one. Data were tested for significance using Student’s t-test. The effect of AURA siRNA treatment on Acet.tub and α-tub protein levels was found to be not significant (n.s.), whereas the treatment had a significant effect on GLI2 (FL), *P* < 0.01 (**). DAPI, 4‘,6-diamidino-2-phenylindole; IFM, immunofluorescence microscopy; OSE, ovarian surface epithelium; WB, western blot.

To further explore a possible link between AURA and lack of cilia in cancer OSE cells, we investigated whether knockdown of AURA by siRNA affected the frequency of ciliated SK-OV3 cells during growth arrest. Figure 7A shows results from three successful knockdowns of AURA, as judged by WB analysis. This analysis also demonstrated that knockdown of AURA does not affect the ability of cells to enter growth arrest upon 72 hours of serum depletion, visualized by the even protein level of p-RB in mock and AURA siRNA-treated cells (Figure 7A, top panels). The bar graph representations in Figure 7A show the percentages of primary cilia in the three SK-OV3 cultures with and without knockdown of AURA, quantified by IFM analysis. Taken together, the data show a modest, but significant and about two-fold increase in the number of ciliated cells in the AURA-depleted cultures relative to mock-transfected control cells (Figure 7B). Lastly, WB analysis demonstrated that knockdown of AURA in SK-OV3 cells results in reduced cellular level of GLI2 full-length activator (Figure 7C,D), presumably due to the higher frequency of primary cilia in these cultures. In contrast, knockdown of AURA had no detectable effect on the level of acetylated α tubulin, suggesting that this type of acetylation is not associated with AURA activity in OSE cells (Figure 7C,D).

## Discussion

In this study, we investigated the occurrence of primary cilia in human wt and cancer OSE cells with a focus on the correlation between AURA and the presence or absence of primary cilia with functional Hh signaling and expression of PDGFRα. Our results show that EOC cells are mostly devoid of primary cilia, and we suggest that this in part may be due to increased expression of AURA in these cells. These findings are in agreement with other studies on cancer cells, such as pancreatic adenocarcinoma cells, basal cell carcinoma cells, and clear cell renal cell carcinomas that also have reduced frequency of primary cilia [44,46,83,84], which in some cases can be explained by over expression of AURA [83]. However, in contrast to, for example, pancreatic cancer cells that may not form cilia because the cells fail to enter growth arrest [84], cancer OSE cells, such as SK-OV3, enter growth arrest upon serum depletion at a level comparable to that of wt OSE cells. The vast majority of OVCAR3 cells also entered growth arrest upon serum depletion, although not as many as SK-OV3 cells. Thus, lack of cilia in these cells seems not to be caused merely by a failure of the cells to become quiescent, suggesting that ovarian cancer cells have defects in the regulatory proteins that control ciliary assembly and/or disassembly.

How is ciliary formation perturbed in cancer OSE cells? Initially, we investigated the expression and localization of two IFT proteins, IFT20 and IFT88, essential for the assembly of primary cilia and found no obvious difference between normal human OSE cells and the two ovarian cancer cell lines. In contrast, we observed a dramatic decrease in the expression of AURA in growth-arrested wt OSE cells compared to growth-arrested SK-OV3 and OVCAR3 cells. Although SK-OV3 and OVCAR3 cells differ, for example in regard to morphology and ability to enter growth arrest, both cell lines maintained a high level of AURA at centrosomes in cells not forming primary cilia. Since AURA has been implicated in ciliary disassembly [54], we suggest that high levels of AURA at the centrosomal region suppress ciliary formation and/or promote ciliary disassembly in growth-arrested cancer OSE cells. This may have dire consequences for regulation of signaling pathways that are coordinated by primary cilia such as Hh and PDGFRα signaling,which, when aberrantly regulated, are associated with EOC [7-14]. Indeed, we here show that PDGFRα and essential components of the Hh pathway, including SMO, PTCH1 and GLI2, localize to primary cilia of wt OSE cells and that cancer OSE cells display increased basal expression of Hh responsive genes. Further, in cancer OSE cells, there is a defect in expression and/or glycosylation of PDGFRα, in that SK-OV3 cells are not up-regulating PDGFRα expression during growth arrest, and that both up-regulation and glycosylation of the receptor is hampered in OVCAR3 cells. Previously, up-regulation of PDGFRα during growth arrest was shown to be blocked in *Tg737*^*orpk*^ mouse embryonic fibroblasts, which have a hypomorphic mutation in IFT88 and, therefore, form no or very short primary cilia [74]. We suggest that defects in ciliary formation due to over-expression and centrosomal localization of AURA in cancer OSE cells in a similar way may perturb proper Hh signaling as well as PDGFRα expression and function leading to homeostatic imbalance of the ovarian surface epithelium.

In order to investigate AURA function in the formation of primary cilia in more detail, we conducted siRNA knockdown of AURA in growth-arrested SK-OV3 cells, since these cells entered growth arrest upon serum depletion at a level comparable to that of wt OSE cells. AURA knockdown increased the number of ciliated cancer OSE cells albeit to a small, but significant, extent, and this was accompanied by a lower level of the full-length activator form of the GLI2 protein, involved in Hh signaling. These results are similar to previous results reported for, for example, renal cancer cells that lack the von Hippel-Lindau tumor suppressor protein; in these cells, it was shown that siRNA-mediated inhibition of the HEF1-AURA pathway caused a significant increase in the frequency of ciliated cells, whereas over expression of AURA or HEF1 in control renal cells promoted cilia loss [83]. Thus over-expression of AURA and loss of primary cilia may be a common characteristic of several types of cancers, in which a moderate restoration of ciliary formation is associated in part with a reduction in aberrant Hh signaling. The fact that AURA siRNA did not fully restore ciliary formation in cancer OSE cells, suggests that the cells were not completely depleted for AURA and/or that the function of other regulatory proteins in ciliary assembly and maintenance is disrupted.

A number of proteins have been suggested to play a role in regulating AURA activity and/or expression. A prominent example is the tumor suppressor protein, CHFR, which has been implicated in multiple human cancers, including EOC [19,85,86]. Originally, CHFR was shown to function as a mitotic checkpoint protein required for tumor suppression, partly through ubiquitination and targeting of AURA for degradation in the proteasome [31,32,87]. In concurrence with previous findings that CHFR localizes at centrosomes in interphase cells [81] and at spindle poles in mitotic cells [82], we found that CHFR localizes to the centrosomal region at the base of primary cilia and in some cases along the length of the cilium in growth-arrested OSE and RPE cells. This is the first report on the localization of this tumor suppressor protein to primary cilia, and although speculative at this point, we suggest that CHFR may function at the cilium to promote cilia stability through inactivation of AURA.

Several proteins are known to interact with AURA during mitosis, but AURA partners and downstream targets at other cell cycle stages are less investigated. In a seminal work by Pugacheva *et al*. [54] it was shown that ciliary disassembly in RPE cells is in part coordinated by AURA-mediated activation of HDAC6, a tubulin deacetylase that promotes destabilization of microtubules [88-90]. In contrast, Sharma *et al*. [91] used the same cell type to show that inhibition of HDAC6 followed by increased level of microtubule acetylation did not affect cilia stability in concurrence with the findings that HDAC6-deficient mice are viable and have no phenotypes associated with known ciliopathies [92]. Similarly, we find that knockdown of AURA by siRNA did not affect the overall level of acetylated tubulin as judged by WB analysis, suggesting that tubulin deacetylase(s) is not the major target for AURA-induced ciliary disassembly or inhibition of ciliary assembly in OSE cells.

## Conclusions

In this work we have established a new platform from which to investigate cellular processes and signaling pathways in ovarian cancer using primary cultures of human OSE cells as well as cultures of human ovarian cancer cell lines. We show that EOC, which comprises the vast majority of human ovarian cancers, is associated with defects in formation of primary cilia that control signaling pathways in ovarian homeostasis such as Hh and PDGFRα signaling. We also show that reduced frequency of primary cilia in cancer cells correlates with overexpression of AURA and persistent localization of AURA to the centrosome in growth arrested cells devoid of primary cilia. We further show that the tumor suppressor protein, CHFR, which inactivates AURA and when mutated or expressed at low levels causes ovarian tumorigenesis, is a centrosomal protein that localizes to the ciliary base in growth arrested wt OSE cells. Future analysis should focus on how CHFR and AURA interact at the primary cilium to control downstream targets in ciliary assembly, disassembly and function.

## Methods

### Collection of human ovaries and tissue sectioning

Healthy human ovaries were sent to the Laboratory of Reproductive Biology at the University Hospital of Copenhagen for cryopreservation (Cryopreservation of ovarian tissue has been approved by the Minister of Health in Denmark and by the ethical committee of the municipalities of Copenhagen and Frederiksberg, journal number KF/01/170/99) from women about to initiate chemotherapy for malignancies other than ovarian cancer. Tissue specimens were dissected into appropriate tissue blocks and fixed for 12 to 24 hours at 4°C in Bouin’s fixatives. The specimens were dehydrated with graded alcohols, cleared in xylene, and embedded in paraffin wax. Serial sections, 5 μm thick, were cut and placed on silanized glass slides. Representative sections of each series were stained with H & E.

### Cell cultures

OSE cells were obtained by scraping the surface of the ovaries with a surgical blade as described elsewhere [60]. The cells were collected in Iscove’s modified Dulbecco’s medium (Invitrogen, Taastrup, Denmark) with 1% penicillin-streptomycin (Invitrogen), immediately followed by centrifugation at 300 x *g* for five minutes at room temperature. The cell pellet was resuspended in OSE growth medium (Minimum Essential Medium α-medium [Invitrogen], 15% fetal bovine serum [FBS; Invitrogen], 1% Glutamax™-1 [Invitrogen], 1% Minimum Essential Medium non-essential amino acids solution [Invitrogen], 1% insulin-transferrin-selenium supplement [Invitrogen], 1% penicillin-streptomycin, and 3.3 mU/ml follicle-stimulating hormone/luteinizing hormone (Menopur, Ferring, Kiel, Germany), and placed in a 35-mm culture dish coated with 0.1% gelatin (Sigma, St. Louis, Missouri, USA). The cultures were incubated at 37°C in 5% CO_2_ in air and left undisturbed for at least 48 hours. Medium was changed at intervals of two to three days. The ovarian cancer cell lines OVCAR3 (ATCC-HTB-161) and SK-OV3 (ATCC-HTB-77) were purchased from the American Type Culture Collection (Manassas, Virginia, USA). The cancer cell lines were cultured in OSE growth medium on a gelatin coating as described above. Passing of cells was performed by trypsination. The cell lines were maintained by passaging continuously on a weekly basis. Cells were examined at a sub-confluent stage in the presence of serum (0 hour or interphase cells) or at confluency followed by serum starvation for indicated time periods to induce growth arrest.

Culture, transfection, and selection of stable hTERT-RPE1 cells expressing GFP-CHFR was performed essentially as described [93]. For generation of GFP-CHFR expressing cells, plasmid pEGFP-C1 (Clontech, Mountain View, California, USA) containing full-length *CHFR* coding sequence (kind gift from Kenneth B. Schou, Danish Cancer Society, Copenhagen, Denmark) was used. The culture of NIH3T3 cells was done as described previously [74].

### Immunohistochemical (IHC) analysis

Tissue sections were dewaxed, rehydrated and washed in PBS as previously described [56], followed by rinsing with blocking buffer (5% BSA in PBS) for 15 minutes before incubation with primary antibodies overnight at 4°C. See Table 1 for applied primary antibodies in IHC analysis. The sections were then washed three times in blocking buffer, incubated for 45 minutes in dark with fluorochrome-conjugated secondary antibodies (Alexa Flour 488 and Alexa Flour 568, Invitrogen) diluted 1:600 in blocking buffer,and, after a wash in blocking buffer, briefly incubated with DAPI (Invitrogen, 1:600) in blocking buffer. After washing in PBS, sections were mounted in anti-fade mounting solution (80% glycerol and 2% N-propylgallate in PBS), covered with a glass slide and sealed with nail polish. Differential interference contrast microscopy (DIC) and fluorescence microscopy analysis were carried out with a Nikon Eclipse E600 and an Olympus BX63 microscope. Image acquisition was performed using an Optronics MagnaFire CCD camera and Olympus DP72 color CCD camera. The images were processed for publication in Adobe Photoshop version 6.0.

**Table 1:** Primary antibodies applied in this study

**Antigen**	**IgG**	**Source**	**IHC**	**IFM**	**WB**
Acetylated α-tubulin	mMouse	Sigma	1:1000	1:5000	1:2500
Aurora A/AlK (1 G4)	mRabbit	Cell Signaling		1:100	1:200
Centrin-2 (N-17)	pGoat	Santa Cruz		1:500	
CHFR	pRabbit	This study		1:500	1:10.000
Cytokeratin-8 (M-20)	mMouse	Santa Cruz	1:100	1:100	
Cytokeratin-18	mRabbit	Epitomics	1:100	1:100	1:1500
Detyrosinated α-tubulin	pRabbit	Abcam	1:800	1:800	
EB1 (KT51)	mRat	Absea			1:1000
EB3 (KT36)	mRat	Absea		1:200	
E-cadherin	pRabbit	Cell Signaling	1:25	1:25	1:200
GFP	mMouse	Abcam		1:200	1:1000
IFT20	pRabbit	kind gift from Greg Pazour		1:1000	1:1000
IFT88	pRabbit	kind gift from Greg Pazour		1:1000	1:1000
Ki67	pRabbit	Abcam		1:500	
N-cadherin	pRabbit	Abcam	1:200	1:200	1:800
Patched-1	pGoat	Santa Cruz		1:200	
PCNA (PC10)	mMouse	Cell Signaling			1:2000
Pericentrin (C-16)	pGoat	Santa Cruz		1:200	
Phospho-RB (Ser807/811)	pRabbit	Cell Signaling			1:200
Smoothened	pRabbit	Abcam		1:100	
GLI2 (G-20)	pGoat	Santa Cruz			1:200
GLI2 (H-300)	pRabbit	Santa Cruz		1:100	
Vimentin	mMouse	Abcam	1:100	1:100	1:500
α-tubulin	mMouse	Sigma		1:500	1:2000
β-actin	mMouse	Sigma			1:10.000

### IFM, immunofluorescence microscopy; IgG, immunoglobulin G; IHC, immunohistochemistry; WB, western blot

#### Immunofluoresence microscopy (IFM) analysis

Cells were grown on 12-mm sterile HCl-cleansed coverslips coated with 0.1% gelatin. The coverslips were washed in ice cold PBS and fixed with either 4% paraformaldehyde (PFA; PFA-fix), 4% PFA and methanol (PFA + MeOH-fix) or with 3% PFA in Brinkley Reassembly Buffer 80 (and methanol (mix-fix). For PFA-fix, cells were fixed for 15 minutes at room temperature, washed twice in PBS, and then permeabilized with 0.2% Triton X-100 and 1% BSA in PBS for 12 minutes. For PFA + MeOH-fix, cells were first fixed with 4% PFA for 10 minutes, washed twice in PBS, and fixed again for 5 minutes in ice-cold methanol. After removal of the methanol, the coverslips were allowed to air dry for a short period followed by permeabilization with 0.2% Triton X-100 and 1% BSA in PBS for 12 minutes. For mix-fix, cells were fixed with 3% PFA in Brinkley Reassembly Buffer 80 (80 mM PIPES pH 6.9, 1 mM EGTA, 1 mM MgCl_2_) for two minutes, washed in ice cold PBS, and fixed again for two minutes in ice-cold methanol. After removal of the methanol, the coverslips were allowed to air dry for a short period followed by rehydration in PBS. To avoid unspecific antibody binding, coverslips (all kinds of fixation and permeabilization) were incubated in blocking buffer (2% BSA in PBS) for 30 minutes at room temperature or overnight at 4°C before transfer to a moisture chamber. The coverslips were subsequently incubated with primary antibodies diluted in blocking buffer for 90 minutes at room temperature or overnight at 4°C (see Table 1 for list of primary antibodies used) followed by 4 x 5 minutes wash in blocking buffer and incubation in dark for 45 minutes with fluorochrome-conjugated secondary antibodies (Alexa Flour 350, Alexa Flour 488, and Alexa Flour 568, all from Invitrogen) diluted 1:600 in blocking buffer. Staining of F-actin with rhodamine-coupled phalloidin (Invitrogen, 1:100) was done concomitantly with secondary antibody incubation. Hereafter, coverslips were washed once in blocking buffer and briefly incubated with DAPI. After washing in PBS, coverslips were mounted on microscope slides in anti-fade mounting solution, sealed with nail-polish and analyzed by microscopy as described for IHC. Cilia frequency was determined by quantifying the number of ciliated and non-ciliated cells of a minimum number of 130 cells for each sample in at least three replicates.

### SDS-PAGE and western blot analysis

SDS-PAGE and WB analysis was carried out essentially as previously described [94]. Cell lysates were prepared in boiling 0.1% SDS lysis buffer supplemented with EDTA-free protease inhibitor cocktail (Roche, Mannheim, Germany) and 1 mM Na_3_VO_4_. Lysates were sonicated and centrifuged to precipitate cell debris, and protein concentrations were measured using a DC Protein Assay (Bio-Rad, Hercules, California, USA) according to the manufacturer’s instruction. Proteins were separated under reducing and denaturing conditions by SDS-PAGE) using 10% Bis-Tris precast gels (Invitrogen) followed by electrophoretic transfer to nitrocellulose membranes (Invitrogen). Membranes were incubated for at least 30 minutes at room temperature or overnight at 4°C in 5% nonfat dry milk in Tris Buffered Saline with Tween (5% milk-TBST; 10 mM Tris–HCl (pH 7.5), 120 mM NaCl, 0.1% Tween 20), before incubation with primary antibodies for two hours at room temperature or overnight at 4°C in moisture chambers (see Table 1 for antibodies used in WB analysis). Antibodies were diluted in 5% milk-TBST as indicated below. Membranes were washed several times in TBST followed by incubation with alkaline phosphatase-conjugated secondary antibodies (Sigma) in 5% milk-TBST for 45 minutes at room temperature. Blots were washed in TBST and protein bands were visualized using BCIP/NBT Phosphatase Substrate (KPL, Gaithersburg, Maryland, USA). After air drying, the developed blots were scanned and processed with Adobe Photoshop version 6.0.

### PCR and primers

In order to monitor the effect of serum starvation on gene expression at the mRNA level RT-PCR was conducted on wt and cancer OSE cells. Cells were grown in culture dishes coated with 0.1% gelatin and serum depleted as described above. RNA was purified using the Nucleospin® RNA II kit (Machery Nagel, Düren, Germany) according to the manufacturer’s instructions. RNA concentrations were determined using a GeneQuant pro spectrophotometer at 260 nm. The RNA samples were stored at −80°C until use. First-strand cDNA was synthesized in 40 μl reactions according to instructions by Invitrogen using SuperScript™ II Reverse Transcriptase (400 units, Invitrogen), 250 ng random primers (Invitrogen), recombinant RNasin® Ribonuclease inhibitor (Promega, Madison, Wisconsin, USA) and 1 μg total RNA; the cDNA samples were stored at −20°C until use. PCR experiments were conducted in 25 μl reactions according to instructions by Promega using the Go Taq® DNA polymerase (Promega), 1 μg cDNA and 0.5 μM of each primer (Table 2). GAPDH was used as an internal control. Annealing temperature and number of amplification cycles were optimized for each primer set. For each primer set-up one negative control without template was included to ascertain the absence of primer-dimer and genomic DNA amplifiation. After amplification, the PCR products were separated by electrophoresis in a 2% agarose gel containing ethidium bromide and visualized with UV light. Images were obtained in a MultiDoc-It digital imaging system (AH Diagnostis, Denmark).

**Table 2:** Primers applied in this study

**Gene**	**Direction**	**Primer sequence (5′ → 3′)**
AURA (from [51])	Forward	GCTGGAGAGCTTAAAATTGCAG
	Reverse	TTTTGTAGGTCTCTTGGTATGTG
GAPDH	Forward	GAAGGTGAAGGTCGGAGTC
	Reverse	GAAGATGGTGATGGGATTTC
GLI1	Forward	GAACCCTTGGAAGGTGATATTC
	Reverse	GGCAGTCAGTTTCATACACAGAT
PTCH1	Forward	ATCAGCCAGTTGACTAAACAG
	Reverse	GTTTCAGGCATGTAGTCGG

### siRNA

AURA knockdown in SK-OV3 cells was performed using ready-to-use custom synthesized siRNA (Thermo Scientific, Lafayette, Colorado, USA) against human AURA (target sequence: GAACUUACUUCUUGGAUCA) or scrambled oligonucleotides (mock) with similar GC content, both at 50 nM, and DharmaFECT transfection reagent (Thermo Scientific) according to the manufacturer’s instructions. Cells were transfected at 60% confluency. The day after siRNA treatment, the cells were serum depleted as described above and were used for experiments 72 hours after siRNA treatment. Cilia quantifications were always accompanied by parallel WBs against AURA to verify knockdown.

### CHFR antibody production

For production of rabbit polyclonal antibodies specific for human CHFR, a maltose binding protein (MBP)-CHFR fusion protein was produced in *Escherichia coli*. The sequence corresponding to the entire coding region of CHFR (Genbank ID AF170724.1) was amplified by PCR from plasmid pEGFP-C1 (Clontech) containing full-length *CHFR* coding sequence (kind gift from Kenneth B. Schou, Danish Cancer Society, Copenhagen, Denmark) using forward (5′-CAGAATTCATGGAGCGGCCCGAG-3′) and reverse (5′-AAGGTCGACTTAGTTTTTGAACCTTGTCTG-3′) primers with recognition sites for *Eco*RI and *Sal*I, respectively, and Herculase DNA polymerase from Stratagene (La Jolla, California, USA). The PCR product was purified and cloned into pMalC2 (New England Biolabs, Ipswich, Massachusetts) using standard procedures and the ligated DNA transformed into competent *E. coli* DH10α cells. Resulting plasmids were control sequenced by Eurofins MWG Operon. Production and purification of MBP-CHFR fusion protein was carried out essentially as described previously [95] and purified MBP-CHFR fusion protein used for polyclonal rabbit antibody production by Yorkshire Bioscience Ltd (Heslington, York, United Kingdom). The resulting CHFR rabbit antiserum was stored in saturated ammonium sulfate solution at 4°C until use.

### Statistical analysis

All experiments were repeated three or more times and data are presented as representative individual experiments or as mean values plus SD. The data were tested for significance using one-way analysis of variance (ANOVA) or Student’s t-test. The level of significance was set at *P* < 0.05 (*), *P* < 0.01 (**), *P* < 0.001 (***).

## Abbreviations

Acet.tub, acetylated α-tubulin; ANOVA, analysis of variance; AURA, aurora A kinase; BSA, bovine serum albumin; CHFR, checkpoint with forkhead-associated and ring finger domains; CK18, cytokeratin 18; CK8, cytokeratin 8; CT, centrin 2; DAPI, 4′,6-diamidino-2-phenylindole; DIC, differential interference contrast microscopy; EOC, epithelial ovarian cancer; FBS, fetal bovine serum; GFP, green fluorescent protein; Glu.tub, detyrosinated α-tubulin; Hh, hedgehog; IFM, immunofluorescence microscopy; IFT, intraflagellar transport; IgG, immunoglobulin G; IHC, immunohistochemistry; LM, light microscopy; MBP, maltose binding protein; OSE, ovarian surface epithelium; PBS, phosphate buffered saline; PCNA, proliferating cell nuclear antigen; PCTN, pericentrin; PDGF, platelet-derived growth factor; PDGFR, platelet-derived growth factor receptor; PFA, paraformaldehyde; p-RB, phosphorylated retinoblastoma protein; PTCH1, patched-1; RT-PCR, reverse transcriptase polymerase chain reaction; SMO, smoothened; TRIS, tris buffered saline with tween; WB, western blot; wt, wild type; α-tub, α-tubulin.

## Competing interests

The authors declare that they have no competing interests.

## Authors’ contributions

This work was carried out in collaboration between all authors. STC, AGB and LBP defined the research theme and designed methods and experiments in collaboration with all authors. DLE carried out the majority of the laboratory experiments, including characterization of ovarian tissue sections and OSE cell cultures by IHC, IFM and WB analyses, investigation of ciliary frequency, analysis of cell cycle status, PCR and WB analysis on the expression of Hh signaling components and IFM and WB analysis on the expression and localization of PDGFRα in OSE cells. AA participated in the collection of primary OSE cultures and carried out IFM studies on the localization of PTCH1, SMO and GLI2 in OSE cells. RM and DLE investigated the localization and expression of IFT proteins in OSE cell cultures by IFM and WB analysis, and DLE and LS performed AURA knock-down analyses. TSJ cloned, affinity-purified, and tested the CHFR antibody in RPE cells by WB and IFM analysis, and ML and DLE carried out localization studies on AURA and CHFR in OSE cells by IFM analysis. All authors contributed to data collection, their interpretation and presentation, and all authors have contributed to, seen and approved the manuscript. STC, LBP and DLE were the major contributors to writing up the initial draft of the manuscript.

## Supplementary Material

Additional file 1 **Figure S1.**Characterization of polyclonal antibody against human CHFR WB analysis of lysates from hTERT-RPE1 cells serum-starved for 48 hours (**A**, **B**), or NIH3T3 cells serum-starved for 24 hours (**B**). The generated polyclonal rabbit antibody recognizes a single band on the blots equivalent to the predicted size of CHFR (73 kDa for isoforms 1 and 2). In serum-starved hTERT-RPE1 cells stably expressing GFP-CHFR, the antibody recognizes both endogenous CHFR and exogenous GFP-CHFR (**C**). **D**, **E**) IFM analysis of endogenous CHFR (**D**) and exogenous GFP-CHFR (**E**) in hTERT-RPE1 cells serum-starved for 48 hours and fixed with mix-fix (**D**) or PFA-fix (**E**) (see Methods for details). Anti-acetylated α-tubulin (Acet.tub) was used to detect primary cilia (arrows). In (**D**) localization of endogenous CHFR is visualised with anti-CHFR, whereas exogenous GFP-CHFR is detected with primary antibodies against GFP. Click here for file
