# Supplementary figures and images for: Primary cilia and aberrant cell signaling in epithelial ovarian cancer
Source: Cilia. 2012 Aug 10;1:15. doi: 10.1186/2046-2530-1-15 (PMC3555760; doi:10.1186/2046-2530-1-15)

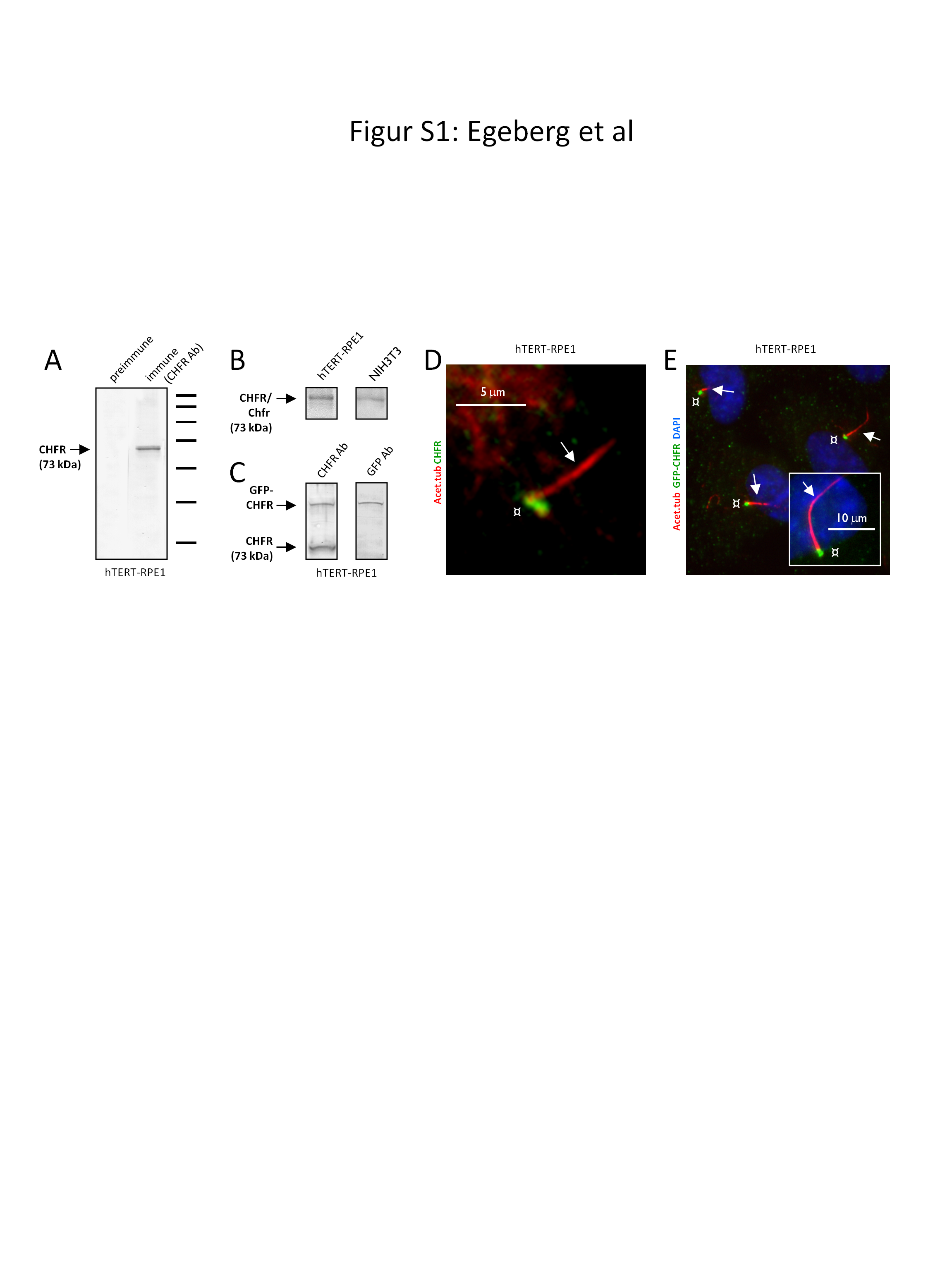

Supplement: Additional file 1 — Figure S1.Characterization of polyclonal antibody against human CHFR WB analysis of lysates from hTERT-RPE1 cells serum-starved for 48 hours (A, B), or NIH3T3 cells serum-starved for 24 hours (B). The generated polyclonal rabbit antibody recognizes a single band on the blots equivalent to the predicted size of CHFR (73 kDa for isoforms 1 and 2). In serum-starved hTERT-RPE1 cells stably expressing GFP-CHFR, the antibody recognizes both endogenous CHFR and exogenous GFP-CHFR (C). D, E) IFM analysis of endogenous CHFR (D) and exogenous GFP-CHFR (E) in hTERT-RPE1 cells serum-starved for 48 hours and fixed with mix-fix (D) or PFA-fix (E) (see Methods for details). Anti-acetylated α-tubulin (Acet.tub) was used to detect primary cilia (arrows). In (D) localization of endogenous CHFR is visualised with anti-CHFR, whereas exogenous GFP-CHFR is detected with primary antibodies against GFP. [file 2046-2530-1-15-S1.tiff]
